# Callosal Influence on Visual Receptive Fields Has an Ocular, an Orientation-and Direction Bias

**DOI:** 10.3389/fnsys.2018.00011

**Published:** 2018-04-16

**Authors:** Sergio A. Conde-Ocazionez, Christiane Jungen, Thomas Wunderle, David Eriksson, Sergio Neuenschwander, Kerstin E. Schmidt

**Affiliations:** ^1^Brain Institute, Federal University of Rio Grande do Norte, Natal, Brazil; ^2^Department of Cardiology and Electrophysiology, University Heart Centre, University Hospital Hamburg-Eppendorf, Hamburg, Germany; ^3^Ernst Strüngmann Institute for Neuroscience in Cooperation with Max Planck Society, Frankfurt, Germany; ^4^Albert-Ludwigs-University of Freiburg, Freiburg, Germany

**Keywords:** interhemispheric connectivity, orientation selectivity, binocular, monocular, anticipation

## Abstract

One leading hypothesis on the nature of visual callosal connections (CC) is that they replicate features of intrahemispheric lateral connections. However, CC act also in the central part of the binocular visual field. In agreement, early experiments in cats indicated that they provide the ipsilateral eye part of binocular receptive fields (RFs) at the vertical midline (Berlucchi and Rizzolatti, 1968), and play a key role in stereoscopic function. But until today callosal inputs to receptive fields activated by one or both eyes were never compared simultaneously, because callosal function has been often studied by cutting or lesioning either corpus callosum or optic chiasm not allowing such a comparison. To investigate the functional contribution of CC in the intact cat visual system we recorded both monocular and binocular neuronal spiking responses and receptive fields in the 17/18 transition zone during reversible deactivation of the contralateral hemisphere. Unexpectedly from many of the previous reports, we observe no change in ocular dominance during CC deactivation. Throughout the transition zone, a majority of RFs shrink, but several also increase in size. RFs are significantly more affected for ipsi- as opposed to contralateral stimulation, but changes are also observed with binocular stimulation. Noteworthy, RF shrinkages are tiny and not correlated to the profound decreases of monocular and binocular firing rates. They depend more on orientation and direction preference than on eccentricity or ocular dominance of the receiving neuron's RF. Our findings confirm that in binocularly viewing mammals, binocular RFs near the midline are constructed via the direct geniculo-cortical pathway. They also support the idea that input from the two eyes complement each other through CC: Rather than linking parts of RFs separated by the vertical meridian, CC convey a modulatory influence, reflecting the feature selectivity of lateral circuits, with a strong cardinal bias.

## Introduction

It has been proposed that visual callosal connections (CC) perpetuate the function of intrahemispheric lateral connections across the two visual hemifields. In agreement, earlier results support the view of an integration of the cortical representation at the vertical midline (VM) (Choudhury et al., 1965; Hubel and Wiesel, 1967; Payne et al., 1991; Makarov et al., 2008; Schmidt et al., 2010).

Due to their specific localization in the brain‘s topography, CC act close to the VM of the binocular visual field of binocular viewing mammals. Accordingly, a role of the corpus callosum in coarse stereoscopic function was suggested for humans (Blakemore, 1970; Mitchell and Blakemore, 1970) and animals with disparity selective neurons (Blakemore, 1969; Lepore and Guillemot, 1982; Gardner and Cynader, 1987).

CC were described to provide the ipsilateral eye part of binocular RFs at the VM (Berlucchi and Rizzolatti, 1968). Early experiments sectioning the corpus callosum or lesioning the contralateral cortex claimed that CC contribute a major part to the binocularity of callosal neurons in cats (striate: Dreher and Cottee, 1975; Payne et al., 1980, 1984; Blakemore et al., 1983; Yinon et al., 1988; extrastriate: Marzi et al., 1980). This result was not confirmed by other studies (Zeki and Fries, 1980; Lepore et al., 1983; Minciacchi and Antonini, 1984; Gardner and Cynader, 1987). Interestingly, anatomical data support the hypothesis that CC preferentially link cortical loci innervated by the temporal retina of the same eye, implying that these fibers do not necessarily contribute to cortical binocularity (Olavarria, 2001).

Most of the studies describing profound RF changes in the absence of callosal input used irreversible sections of the chiasm and/or the corpus callosum. More recent studies of callosal function using reversible deactivation suggested that CC serve to scale responses in a mainly multiplicative manner (Wunderle et al., 2013) acting both directly, changing the receiving neurons' sensitivity to specific features (Schmidt et al., 2010; Altavini et al., 2017), and indirectly, by unspecifically modulating the gain of the receiving neuron (Wunderle et al., 2015). From this type of interaction, CC would be expected to only moderately influence RF size. Further, CC in cats do not interconnect selectively neurons of same or different eye dominance (see also Olavarria, 2001), but of similar orientation (Schmidt et al., 1997; Rochefort et al., 2009), direction (Peiker et al., 2013), and axial selectivity (Rochefort et al., 2009; Schmidt, 2016). Thus, RF properties contributed by callosal input should be observed for both monocularly and binocularly driven responses and reflect the feature selectivity of the anatomical connection.

In order to investigate the functional contribution of callosal connections to monocularly and binocularly activated receptive fields and binocular mechanisms in an intact system we studied spiking responses in the receiving 17/18 transition zone (TZ) during reversible deactivation of the sending hemisphere in cats.

## Materials and methods

### Preparation and surgical procedures

Experiments were carried out in five adult cats bred at the Max Planck Institute's colony and in two animals bred at the colony of Brain Institute of the Federal University of Rio Grande do Norte (UFRN). This study was carried out in accordance with the recommendations of the Society for Neuroscience and with the German Law for the Protection of Animals (Tierschutzgesetz) and the Brazilian Law for the Protection of Animals. The protocol for the first 5 animals was approved by the Regierungspräsidium Darmstadt (Dezernat V54). The protocol for the last two animals was approved by the Ethical Committee for the Use of Animals of the University of Rio Grande do Norte in Natal (CEUA), Centro de Biociencias, UFRN.

Parts of the binocular electrophysiology data were included in a previous study (Peiker et al., 2013).

Anesthesia was induced by an intramuscular injection of ketamine hydrochloride (10 mg/kg Ketamin® 10%, CP-Pharma Partner HGmbH, Burgdorf, Germany), xylazine hydrochloride (1 mg/kg Rompun® 2%, Bayer AG, Leverkusen, Germany) and atropine sulfate (0.1 mg/kg, Atropin®, B. Braun Melsungen AG, Melsungen, Germany). After tracheotomy, we maintained anesthesia with halothane (0.6% for recording, 1.1% for surgery) and a mixture of N_2_O/O_2_ (70/30%). Temperature, heart rate, exhaled CO_2_ level and respiration pressure were monitored continuously.

We performed two rectangular craniotomies of about 8 × 6 mm^2^ centered on Horsley-Clarke coordinates AP 0 to −2, ML +2 symmetrically on both hemispheres, leaving the bone above the superior sagittal sinus intact. On the left hemisphere, the 17/18 border was determined by optical imaging of intrinsic signals after implantation of a titanium recording chamber, applying two different grating stimuli optimized to either the properties of either area 17 (drifting with 0.5 cyc/deg at 4°/s) or area 18 (drifting with 0.15 cyc/deg at 16°/s). The sum of images obtained with stimulation optimal for area 17 was divided by the sum of images obtained with stimulation optimal for area 18. For practical purposes, a zone, which extended 500 μm from the response equality line into either area 17 or 18, was functionally defined as TZ. Electrodes placed within that zone usually recorded from neurons that had RFs within 10° from the VM. This definition corresponds to the definition of the callosal projection zone given by Blakemore et al. (1983), but it should be noted that also neurons beyond that region, especially in area 18, receive callosal input (Segraves and Rosenquist, 1982).

**Figure 1:**
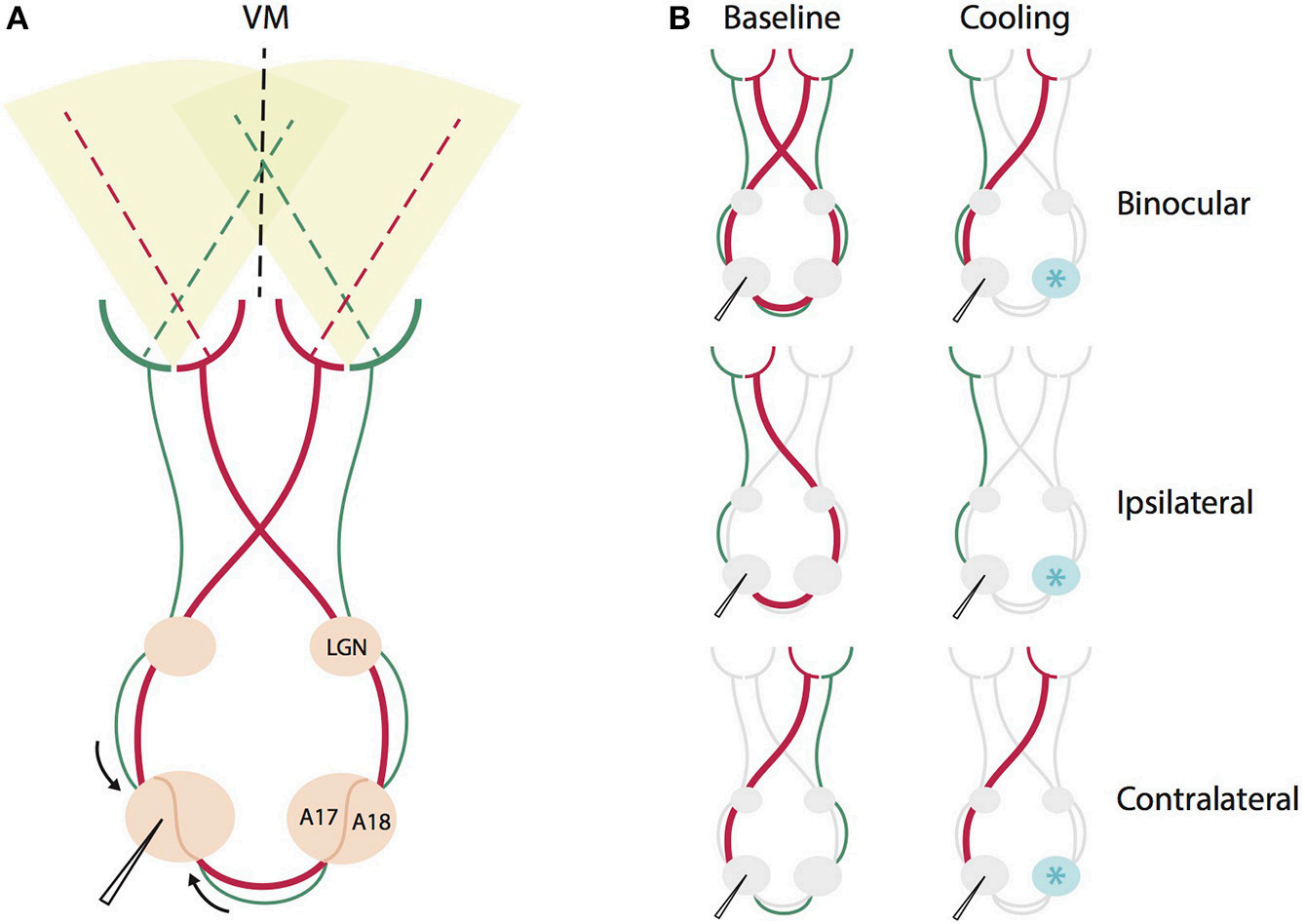
Schematic summary of the direct geniculo-cortical and indirect callosal circuits affected by binocular and monocular stimulation during contralateral cooling deactivation. **(A)** Intact circuit of all direct geniculo-cortical and indirect callosal pathways during binocular stimulation. Red thicker lines indicate pathways crossing in the chiasm to emphasize that this portion of retino-geniculate fibers is larger than the non-chiasm-crossing portion (green). VM: vertical meridian, LGN, lateral geniculate nucleus, A17, area 17, A18, area 18. **(B)** The same circuit for different monocular and binocular stimulations before (left) and during cooling deactivation (right) of the right hemisphere. Cryoloop indicated by a light blue star positioned over the right hemisphere. For binocular stimulation, cooling deactivates both chiasm-crossing and non-chiasm-crossing inputs from the right hemisphere representing the left hemifield. For ipsilateral eye stimulation, cooling deactivates only the larger portion of chiasm-crossing (red) input passing through the corpus callosum. For contralateral stimulation, cooling deactivates only the smaller portion of non-chiasm crossing (green) input passing through the corpus callosum.

On the right hemisphere, a cryoloop was positioned onto the topographically corresponding region (Figure 1, see Cooling Deactivation). At the end of the experiment animals received a lethal dose of pentobarbital sodium (Narcoren, Merial, Germany).

### Eye alignment

For surgical procedures and between recording sessions, the cat's nictitating membranes were closed to prevent the eyes of drying. For recording, pupils were dilated by topical application of atropine sulfate (1%, Atropine-POS, Ursapharm, Germany) and diluted phenylephrin (5% Neosynephrin, Ursapharm, Germany). In order to prevent eye movements, animals were paralyzed by pancuronium bromide (0.3 mg/h, i.v., Pancuronium®, Delta Select GmbH, Pfullingen, Germany) after the surgical preparation steps had been accomplished. Eyes were refracted for a 57 cm viewing distance using correcting contact lenses painted black with a 3 mm artificial pupil. After having defined the left eye's monocular receptive field on the screen, we exactly superimposed the right eye's monocular receptive field by using a prism (Oculus, Germany) in front of the right eye. An enhanced firing rate with binocular stimulation as opposed to the better of the two monocular stimulations confirmed correct superposition. In addition to manual confirmation, we used an automated mapping procedure (Fiorani et al., 2014) to exactly identify receptive field positions and firing rates, for each eye separately. Alignment and refraction were repeatedly checked throughout the experiment.

### Electrophysiological recording

After imaging, the silicon oil was removed and up to 3 Tungsten microelectrode arrays (4 × 4, spacing 250 or 400 mm; 1 M, Microprobes, Gaithersburg, USA) were lowered into the TZ as well as in adjacent central parts of area 17 and 18, for extracellular electrophysiological recordings. After having lowered them by about 800 μm into the cortex the craniotomy was covered with agar and bone wax. After 6 h stabilization time we started to record extra-cellular multi-unit activity and local field potentials using Plexon amplifiers (Plexon Inc., Dallas, 7TX, USA). For multi-unit activity, signals were amplified 1,000 fold, high pass filtered (0.7–6 kHz), thresholded manually around 4 standard deviations well above noise level, digitized with M-series acquisition boards (National Instruments, USA) and stored by a custom-made program (SPASS by Sergio Neuenschwander, in LabView, National Instruments, USA).

### Visual stimulation

Stimuli were presented on a 21″ CRT monitor positioned to cover the central 20° in each visual hemifield in the horizontal and 30° in the vertical.

We determined receptive field position, extent and preferred orientation of all recorded units by presenting whole-field bars moving in 16 different directions (22.5° steps) with a width of 1° and a speed of 20°/s for 2,000 ms as in Fiorani et al. (2014). One recording session consisted of 16 different stimuli, which were presented 20 times to the left, right, or both eyes (= 48 conditions) in a randomized manner using a computer-controlled eye-shutter.

### Cooling deactivation

On the right hemisphere, a surface cryoloop (Lomber et al., 1999) equipped with a temperature sensor touching the cortical surface was positioned above the 17/18 border (Horsley Clarke coordinates AP 0, L −3.5) including the adjacent parts of area 17 and area 18 (Figure 1). We implanted the right hemisphere in all animals with the same probe (5 × 3 mm^2^) for the practical reason of comparability between animals. The probe was covered with clear agar (Agarose type XI, Sigma, Germany) to allow for visual inspection of the cortical surface beneath the probe, and the area was regularly rinsed with saline throughout the experiment. During cooling, chilled methanol (−65°C) was pumped through the cryoploop and the temperature at the sensor was stabilized at 2 ± 1.5°C by regulating the pump velocity. Because cells stop firing at a temperature around 20°C (Lomber et al., 1999), and the temperature gradient is between 10 and 15°/mm (Payne et al., 1991; Lomber et al., 1999, 2002), the fully deactivated cortical region at this temperature is about 8 × 6 mm^2^. Thus, the deactivated area in the contralateral hemisphere did most likely cover not only the TZ, but also a region corresponding in size and extension to the area we are recording from on the ipsilateral hemisphere. This area includes TZ and adjacent parts of area 17 and area 18, spanning 3 mm in medio-lateral direction of the border and 8 mm in AP extent. Comparing to Figure 1 in Olavarria, 2001, the callosally connected region should be largely included in this directly deactivated cortical region.

Recordings were executed before, during and after thermal deactivation (baseline-cooling-recovery). Recording during cooling started after the cryoloop had reached a stable temperature of 2 ± 1.5°C for 5 min. Before recording of the recovery period, a re-warming phase of 20 min was allowed for full recovery of the right hemisphere to baseline temperature and activity level. Units were tested in several cooling cycles with different protocols. Repeated automated mappings of the RFs confirmed their stability and the eye alignment over several protocols. Then, electrodes were moved down, and another recording session started after new units had stabilized.

### Data analysis

In order to obtain single units, we applied *WaveClus*, a spike sorting toolbox developed to determine the spiking times of single units within multi-unit activity. The toolbox calculates a set of parameters based on wavelet decomposition of spike waveforms, followed by a super-paramagnetic clustering (for details see Quiroga et al., 2004).

Single-units had to fulfill the following inclusion criteria: (i) they had to have significantly higher spiking activity to the stimulus than during pre-stimulus time (Wilcoxon rank sum test, *p* < 0.05); (ii) the direction or orientation tuning of the channel reached a certain threshold. We therefore calculated a direction (DSI) and orientation (OSI) selectivity index defined as 1 min the circular variance of the direction or orientation tuning curve, respectively (Swindale, 1998). Those indices range from zero (totally unselective) to one (perfectly selective). Only cells with a DSI or OSI of >0.2 were considered for further analysis; (iii) neurons were classified to belong to the TZ or adjacent area 18 or area 17 according to intrinsic signal imaging prior to electrode implantation (for an example, see Supplementary Figure 3 in Wunderle et al., 2013). This included cells up to eccentricities of 15°; (iv) multi-units produced a circumscribed receptive field for all three mapping procedures, ipsilateral only, contralateral only and binocular; (v) spike rates showed recovery after rewarming.

### Ocular dominance index and contralateral bias index

Based on the single-units' average preferred rate during monocular RF mapping, we calculated an ocular dominance index (ODI) describing each eye's contribution to visually induced neuronal activity during baseline, cooling and recovery:

$$\begin{array}{l} ODI=\frac{FR_{contra}-FR_{ipsi}}{FR_{contra}+FR_{ipsi}};ODI\;\in \left[-1,\;1\right] \end{array}$$

Where *FR*_*contra*_ and *FR*_*ispi*_ stand for the firing rate with contra-and ipsilateral stimulation, respectively. Subsequently, neurons were grouped into five different classes of ocular dominance (simplified from Hubel and Wiesel, 1962). Neurons with an ODI of 1 are driven only by stimulation of the contralateral eye, whereas neurons of ODI class 5 are activated only by stimulation of the ipsilateral eye. ODI class 3 describes neurons that are equally activated by both eyes. Neurons of ODI classes 2 and 4 are preferentially driven by one eye.

We further calculated the contralateral bias index (CBI) modified from Caleo et al. (2007):

$$\begin{array}{l} CBI=\frac{\left(N_{OD1}-N_{OD5}\right)+\frac{1}{2}\left(N_{OD2}-N_{OD4}\right)+N_{Total}\;}{{2*N}_{Total}} \end{array}$$

Where *N*_*ODi*_ stands for the number of neurons in the i-th class. This index describes the bias of the overall ODI distribution toward one eye.

### Receptive field area

A peri-stimulus time histogram (PSTH) of the spiking response to the drifting bar was created (width = 1°, length = 30°, speed = 20°/s) using a Gaussian smoothing kernel with a SD of 12.7 ms for each of the 16 mapping directions. Then, each PSTH was normalized to its maximum height in order to weight each stimulus direction equally. Subsequently, PSTHs for a certain channel were projected to the visual mapping field and summed across stimulus directions. The resulting response density maps were additionally low pass filtered (2D Gaussian smoothing with a SD of 5.88°) and the receptive field was defined as the area above 70% of the maximal response. This was done separately for ipsilateral, contralateral and binocular stimulation.

### Modulation index (MI)

For quantification of cooling associated changes, of the variables ODI, CBI, receptive field size and preferred spike rate we calculated a modulation index between cooling and baseline:

$$\begin{array}{l} MI_{C/R}=\frac{Var_{C/R}-Var_{B}}{Var_{C/R}+Var_{B}} \end{array}$$

Where *Var*_*C*/*R*_ and *Var*_*B*_ stands for a given variable measured during cooling or recovery and baseline recording sessions respectively. Resulting values < 0 refer to smaller values of the variable compared to baseline and values >0 indicate higher values as in the baseline.

## Results

For the analysis of changes in ocular dominance and receptive field size, we considered 141 single units from 5 cats with RFs for both monocular and binocular stimulation during baseline, cooling and recovery, 76 from matrix electrodes placed in the TZ, and, for comparison, 42 from electrodes within the area 17 adjacent to the TZ and 23 from electrodes placed into area 18 adjacent to the TZ. It is important to note that in the cat–though the TZ is the most densely interconnected region-supposedly all these regions receive CC (e.g., Boyd and Matsubara, 1994).

### Ocular dominance distribution

**Figure 2:**
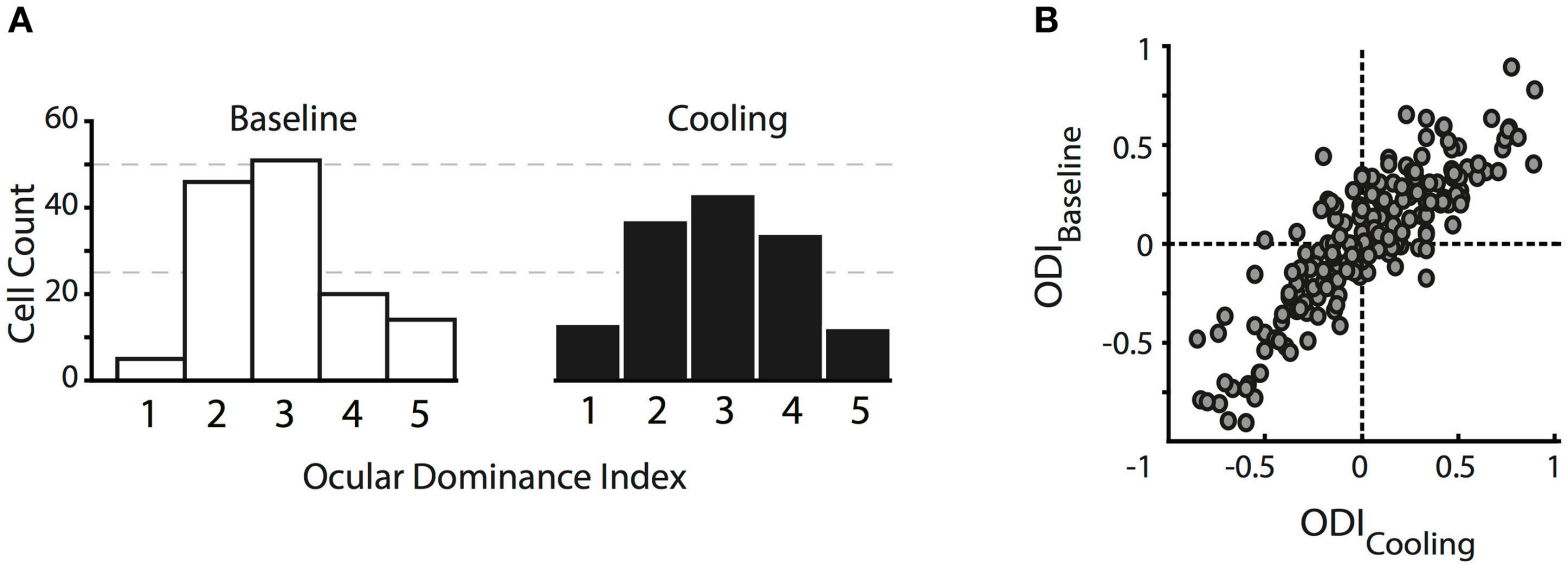
Changes in the ocular dominance distribution. **(A)** Classical ocular dominance response classes adapted from Hubel and Wiesel (1962) based on 5 equidistant intervals of the ocular dominance index. Class 1, contralateral eye only; class 2, dominated by the contralateral eye; class 3, binocularly driven; class 4, dominated by the ipsilateral eye; class 5, ipsilateral eye only. **(B)** original ocular dominance indices during baseline and reversible deactivation of CC. Note that the distributions do not change with absent visual callosal input.

In contrast to previous reports, the ocular dominance (OD) distribution of the investigated cells did not change significantly in the absence of callosal input. We calculated an ocular dominance index (see Methods) ranging from −1 to 1, where +1 stands for contralateral only and −1 for ipsilateral only response. As expected for cat primary visual cortex, the distribution took an inverted U shape with a peak around 0 pointing toward a majority of binocularly driven cells. For illustration, we categorized neurons into five ocular dominance classes (simplified from Hubel and Wiesel, 1962, see Methods) with class 1 including neurons driven by the contralateral eye only, class 2 with neurons dominated by the contralateral eye, class 3 with neurons binocularly driven, class 4 with neurons dominated by the ipsilateral eye, and class 5 including neurons driven by the ipsilateral eye only (Figure 2A).

During deactivation of the contralateral TZ, neurons continued to be driven binocularly and their ocular dominance indices during cooling did not differ significantly from the baseline indices (Figure 2B, Wilcoxon signed rank, *p* = 0.93, *n* = 141). Ocular dominance changes continued to be not significant when separating the indices into the three areas we recorded from, i.e., area 17, TZ or area 18.

Still, there appeared to be a tiny trend of ODs to increase, i.e., enhancing the overall contralateral bias. Therefore, we calculated the contralateral bias index (CBI) per data set, which denotes the bias of the OD distribution toward the contralateral eye in a defined area, a measure commonly used in mice who do not have ocular dominance columns (Caleo et al., 2007). A CBI of 1 stands for total activation of the investigated area through the contralateral eye, whereas a CBI of 0 denotes activity induced by the ipsilateral eye only. The index ranged from 0.1 to 0.9 throughout the six data sets and three different areas, depending on the absolute number of neurons in those zones. The CBI did not change significantly during cooling (*n* = 16, 4 data sets for area 17, 7 for TZ, 5 for area 18, Mann-Whitney U, *p* > 0.5). In conclusion, deactivation of interhemispheric input did not significantly alter the overall ocular dominance distribution in the receiving hemisphere.

### Area size

**Figure 3:**
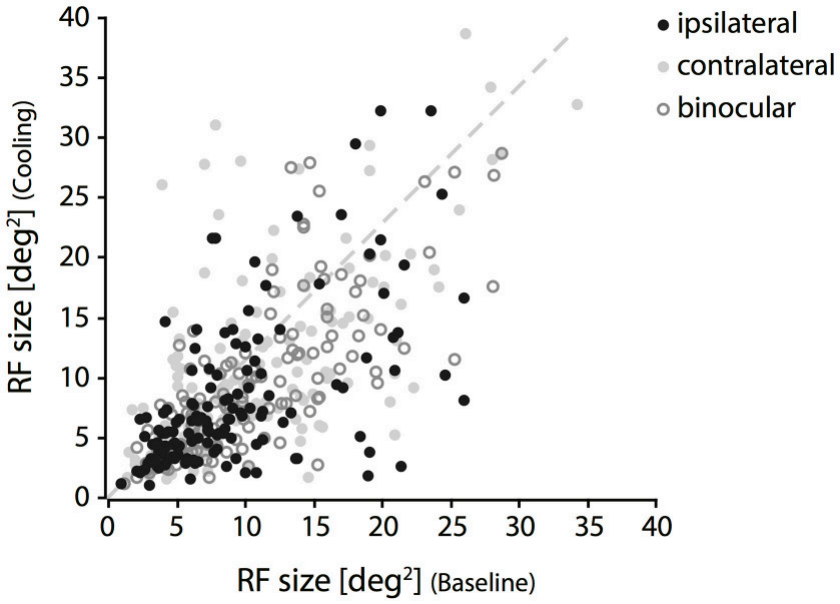
Changes of RF size during deactivation of CC. Receptive field sizes estimated during cooling vs. baseline recordings for binocular (open circles), contralateral (light gray cricles) and ipsilateral (black circles) stimulation. Note that both increases and decreases occur for all three stimulation conditions. RF shrinkage is more frequent with ipsilateral stimulation.

In accordance with the lack of OD change during cooling of the contralateral visual cortex, we also could not reproduce the previously described distinct loss of ipsilateral “halfs” of RFs. Throughout our sample, changes were moderate and affected almost all receptive field locations, not only those close to the VM representation. Out of all 147 RFs, only few fields (*n* = 6) got completely lost during cooling. Although these RFs stem from the subset of TZ neurons, their position was not systematically related to the VM. Among the remaining 141 RFs within the transcallosally interconnected regions and fulfilling our criteria we observed mainly shrinkages, predominantly for the *ipsilaterally* driven RF part (Figure 3, black filled circles). Shrinkage, in the absence of CC, means lack of facilitation. Also, occasional increases occurred during cooling deactivation of the callosal input, frequently for the *contralaterally* driven RF part (Figure 3, gray filled circles). Increase, in the absence of CC, means release from inhibition.

**Figure 4:**
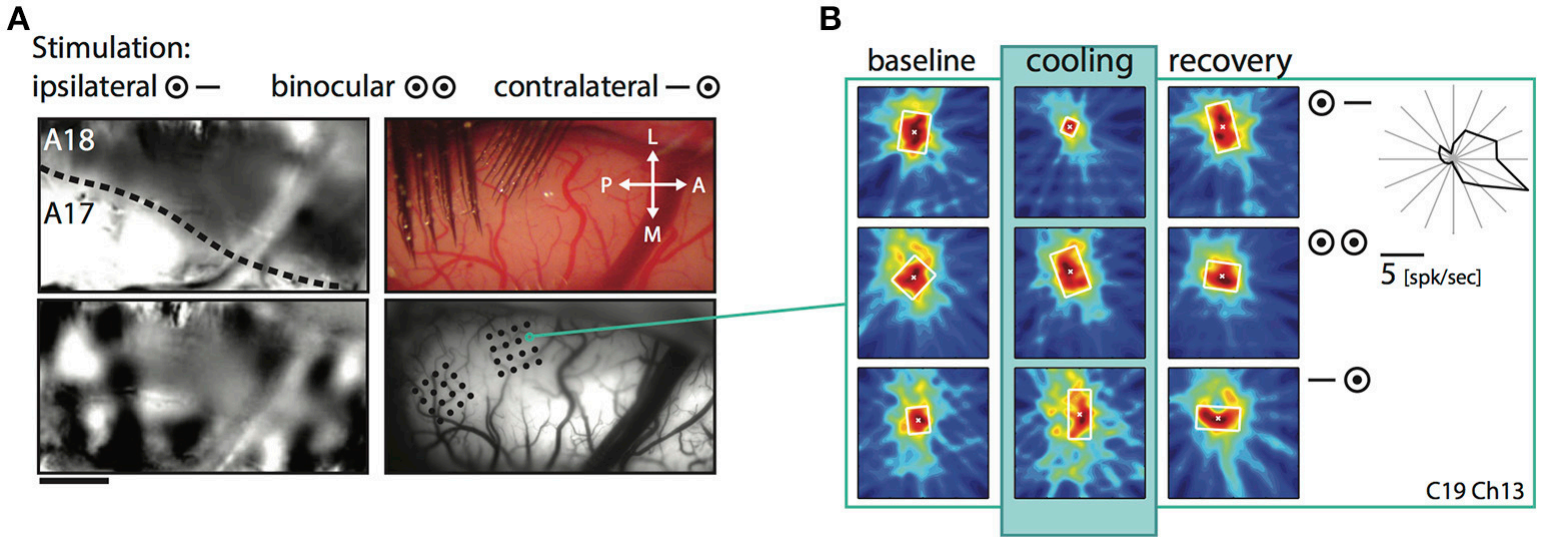
Example receptive field of a TZ unit during CC deactivation for binocular and both monocular stimulations. **(A)** Upper left, intrinsic signal difference image to identify the 17/18 border (see Methods). Dark, optimal activation for area 18; light, optimal activation for area 17; dotted line, estimated area 17/18 border. Upper right, photograph of the recorded area on the left with matrix electrodes in position. Lower left, difference image for orthogonal (horizontal vs. vertical) stimulations. Lower right, vessel image with schematic drawing of the recording sites. **(B)** Receptive field extent of an example unit from the matrix on the area 18 side of the border. Left column, baseline recording, middle column, during cooling deactivation of CC, right column, recovery recording. Rows for the binocular and monocular stimulations as indicated by the symbols. Upper right: Polar plot of the mean firing rate of the unit for 16 different directions.

Shrinkage was homogeneous, because it did not affect a particular part of the RF, but occurred in all investigated regions and depended only on the stimulation. Consistent with previous reports it affected more strongly the part of the response that was driven by the *ipsilateral* eye, especially in the TZ. The example in Figure 4 demonstrates cooling induced changes for an example unit in the TZ situated on the area 18 side of the border during the *binocular, ipsilateral* and *contralateral* stimulation (Figure 4, C19Ch13). The *ipsilaterally* driven RF (middle row) clearly shrinks, whereas the *contralaterally* driven part even slightly increases (lower row) during cooling.

**Figure 5:**
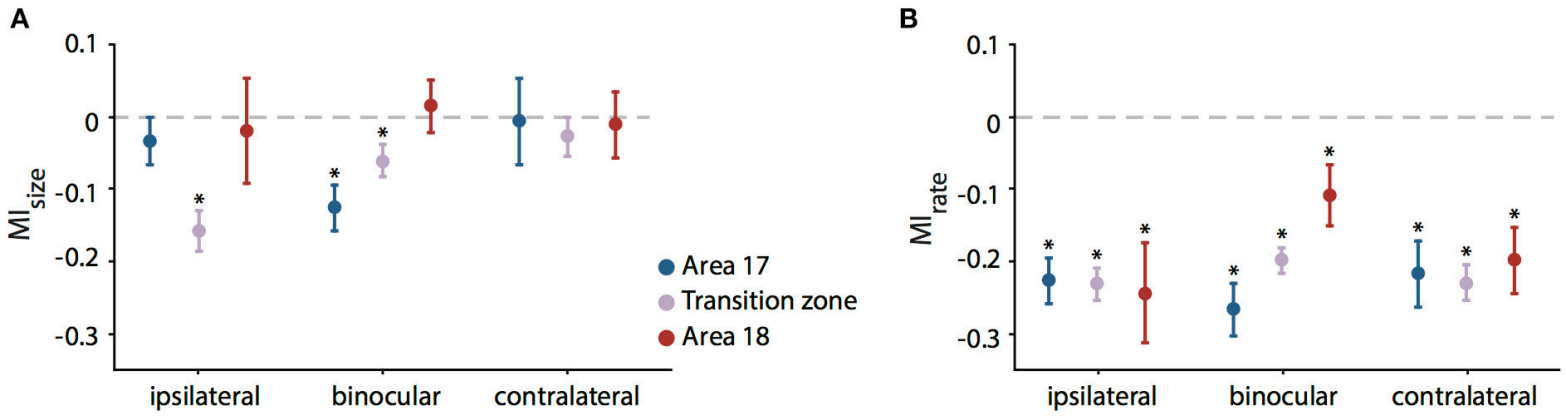
Mean modulation indices for RF size (MI_size_, **A**) and firing rate (MI_rate_, **B**) during CC deactivation. Values are separated for the recorded sub-areas 17, transition zone and 18 (color code on the right) and different eye stimulation (indicated below). Significant decreases (paired *t*-test, see Results) are indicated by stars. Note that units in the transition zone decrease in RF size in particular during ipsilateral stimulation. In contrast, firing rates decrease significantly in all zones and for all stimulations.

On average, the decrease in RF size was significant for the condition *ipsilateral only* (*p* = 0.0015, *n* = 141, Wilcoxon Signed Rank) and for the condition *binocular* (*p* = 0.008, Wilcoxon Signed Rank), but not for the condition *contralateral only* (*p* = 0.33, Wilcoxon Signed Rank). At a closer look, it turned out that for *ipsilateral* stimulation, RFs changed only in the TZ (*p* < 0.0001, *n* = 76, Wilcoxon Signed Rank), whereas for *binocular* stimulation weaker though still significant changes occurred in both area 17 (*p* = 0.0003, *n* = 42, Wilcoxon Signed Rank) and TZ (*p* = 0.024, *n* = 76, Wilcoxon Signed Rank; see Figure 5A, stars).

In order to normalize for the initial size of the receptive field, we used the modulation index as introduced in previous studies for describing the effect of cooling (Wunderle et al., 2013). Accordingly, the average MI (see Methods) for RF size of all 141 units during *ipsilateral* stimulation was −0.1 ± 0.02 (SEM, standard error of the mean), during *binocular* stimulation −0.068 ± 0.017 (SEM) and during *contralateral* stimulation −0.018 ± 0.024 (SEM), indicating only a moderate influence of callosal input on receptive field size as compared to the accompanying rate change. Figure 5 demonstrates the MIs for both RF size (Figure 5A, MI_size_) and firing rate with bar stimulation (Figure 5B, MI_rate_) separated by area and stimulated eye.

To exclude that the pattern of RF changes was a mere consequence of the known firing rate changes when removing callosal input we correlated the MI_size_ with the MI_rate._ However, there was no significant correlation between changes in firing rate and the changes in RF size. Overall, firing rate decreased much more than RF size and, therefore, MIs were lower. With the same bar stimulus applied to examine the RF sizes, average firing rate MIs in the investigated neuron population were −0.23 ± 0.23 for *ipsilateral* (31.8 ± 34% firing rate change), −0.2 ± 0.19 (29.2 ± 27.5%) for *binocular* and −0.22 ± 0.22 (30.3 ± 35.2%) for *contralateral* stimulation showing no difference between different stimulations (Figure 5B).

In area 17, the effect on binocular RFs was quite strong as compared to the individual monocular fields (see Figure 5A). In agreement, the sum of the two monocular effects (MI_ipsi_ + MI_contra_) was significantly smaller than the MI_size_ for binocular stimulation, though with weak effect size (paired *t*-test, *p* = 0.041, *n* = 42). In all other subgroups, effects on the binocularly driven field size were not significantly different from the sum of the monocular effects.

**Figure 6:**
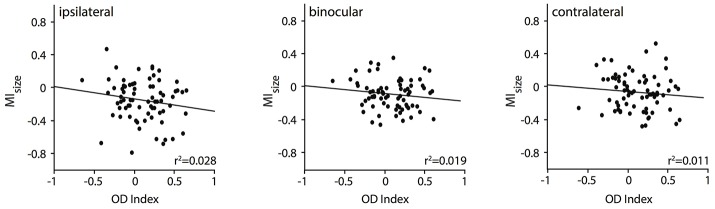
RF changes for binocular and the two monocular stimulations during CC deactivation (MI_size_) are not significantly correlated with the OD index of the callosal input receiving neuron.

So far, we have observed a significant relationship of the RF shrinkage during removal of callosal input with the stimulation, i.e., ipsilaterally and binocularly driven geniculocortical input, and with the area where the receiving neuron is situated, i.e., TZ. This is in agreement with Olavarria (2001) suggesting that in particular ipsilaterally dominated neurons—from TZ adjacent areas 17 and 18, which are included in the deactivated region in our experiment—project to the other transition zone. This author had also proposed that, in the TZ, in particular, contralaterally dominated neurons receive callosal input-from ipsilateral domains outside the TZ. Thus, in a next step, we wanted to explore whether the RF size changes were related to the ocular dominance of the callosal input receiving neurons in the TZ. To this end, we correlated their RF size changes, quantified by the MI values, with their baseline ocular dominance index (*n* = 76 neurons, Figure 6). It seems that neurons dominated by the contralateral eye (positive OI indices) are slightly more affected by ipsilateral (Figure 6, left panel) than by binocular (middle panel) or contralateral input (right panel), which would be predicted by the anatomical data of Olavarria (2001). However, overall, the two measures, MI and OI, were not significantly correlated. Neurons dominated by the contralateral eye (positive OD indices) in the TZ are not affected significantly more than those, which are equally driven by both eyes (around OD index 0) or by the ipsilateral eye only (negative OD indices).

### Influence of the neuron's orientation on receptive field changes-cardinal bias

**Figure 7:**
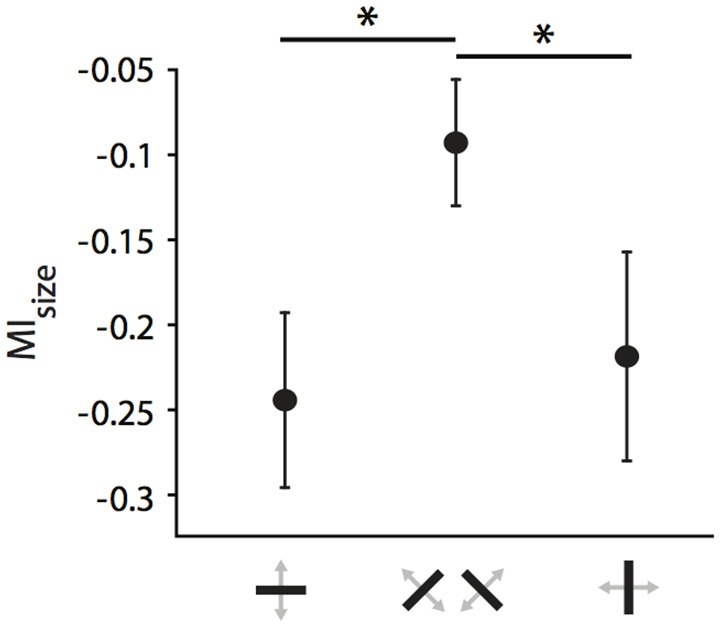
Cardinal bias in the influence of CC on receptive field size in the TZ. Mean modulation indices for RF decrease during ipsilateral stimulation separated by the receiving neuron's orientation preference. Note, that receptive fields of either horizontally or vertically preferring neurons shrink significantly more than those of obliquely preferring neurons when CC are deactivated. Stars indicate significant difference (see Results).

For the following analyses, we focused first on the strongly affected population of neurons receiving callosal input in the TZ. Previously, we had observed that orientation and direction selectivity of these neurons play a role in the strength of the modulatory influence, i.e., that CC act in an orientation- and direction-selective manner (Peiker et al., 2013). As neurons preferring cardinal contours have been shown previously to be more susceptible to visual callosal input (Schmidt et al., 2011; Altavini et al., 2017) we split all RFs under investigation into three categories according to the orientation preference of the neurons (vertical, horizontal, oblique ± 22.5 degrees; Figure 7). We observe that when stimulated *ipsilaterally*, RFs of neurons preferring either *horizontal* or *vertical* (i.e., *cardinal*), but not to *oblique* contours shrink significantly during CC deactivation (n_cardinal_ = 37, Wilcoxon signed rank, *p* = 0.0002; n_oblique_ = 39, *p* = 0.06). There is a “*cardinal* shrinking bias” in the group of *ipsilaterally* driven RFs, but not among the *binocularly* or *contralaterally* driven RFs (data not shown). Accordingly, the MI_size_ of *ipsilaterally* driven RFs are significantly different between the two groups (Figure 7; 37 cardinal vs. 39 oblique, Mann-Whitney-U, p_ipsilateral_ = 0.02; p_binocular_ = 0.33; p_contralateral_ = 0.63).

### Directional bias in the TZ

**Figure 8:**
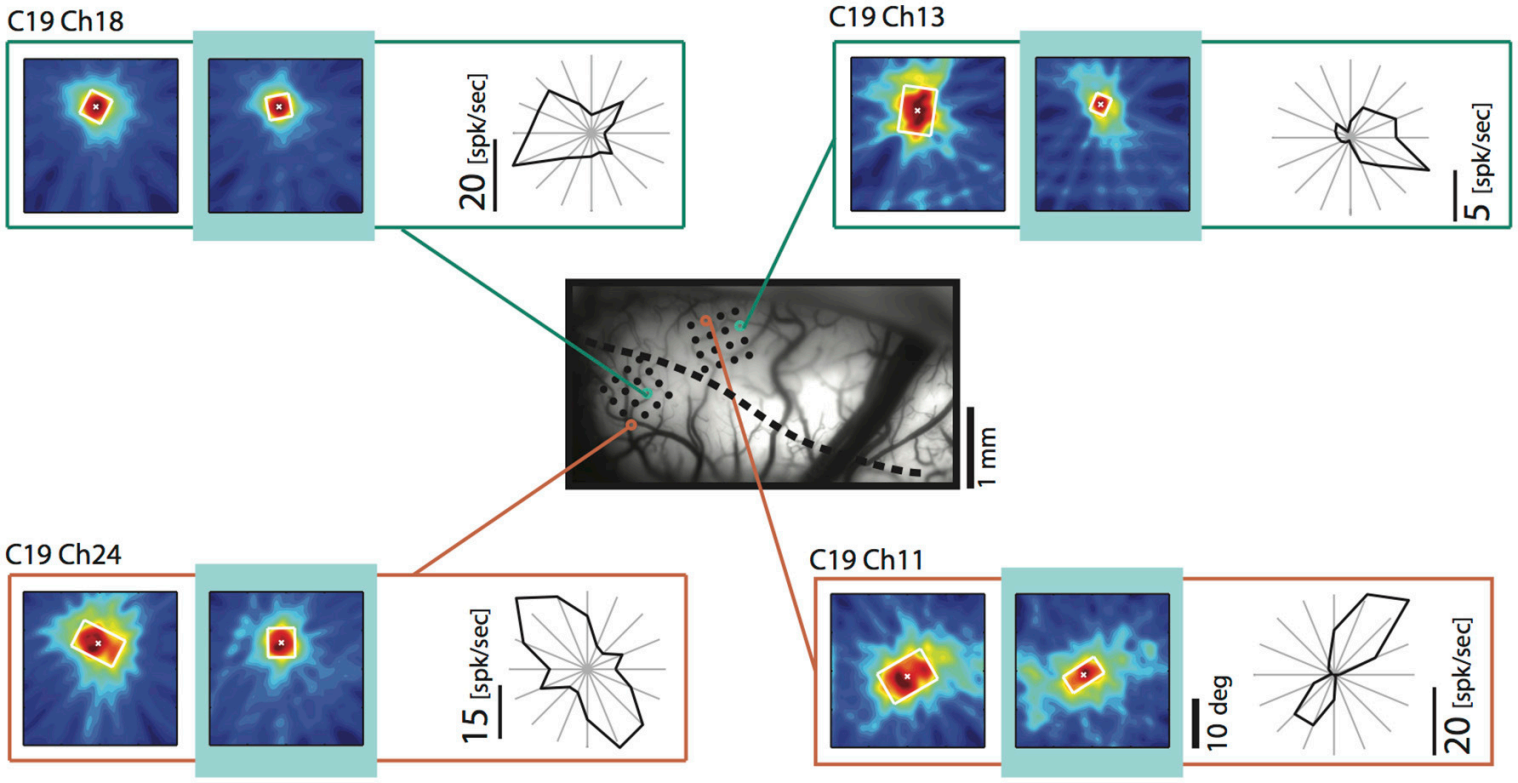
Example receptive fields obtained during ipsilateral stimulation during removal of callosal input (light blue) for neurons preferring movement *into* **(upper left)** and *out of* **(upper right)** the cooled hemifield, as well as *up and down* movement (**lower left** and **right**). Vessel image and conventions as in Figure 4. Note that the neuron preferring the *out of* movement is particularly affected. Neurons preferring horizontal contours decrease RF size independent of their preferred direction of motion and position.

Since we had observed a strong influence of the direction of motion across the vertical meridian and its relation to the side of cooling before (Peiker et al., 2013), we took a closer look on the fields of neurons preferring contours moving across the VM. Figure 8 demonstrates that TZ neurons preferring horizontal contours moving *up* and *down* (Figure 8, chs 11 and 24) and vertical contours moving *out of* the cooled hemifield toward right (Figure 8, ch 13) show shrinkage of their receptive field during removal of callosal input when stimulated ipsilaterally. These fields have in common that the feature preferred by the neuron potentially belongs to either a shape or movement trajectory crossing the vertical meridian from the perspective *out of* the cooled left hemifield. In contrast, a field of a neuron preferring vertical contours moving *into* the cooled hemifield is not affected (Ch 18). This is in agreement with the previous results on optimally driven binocular spiking of cardinally preferring neurons (Peiker et al., 2013).

**Figure 9:**
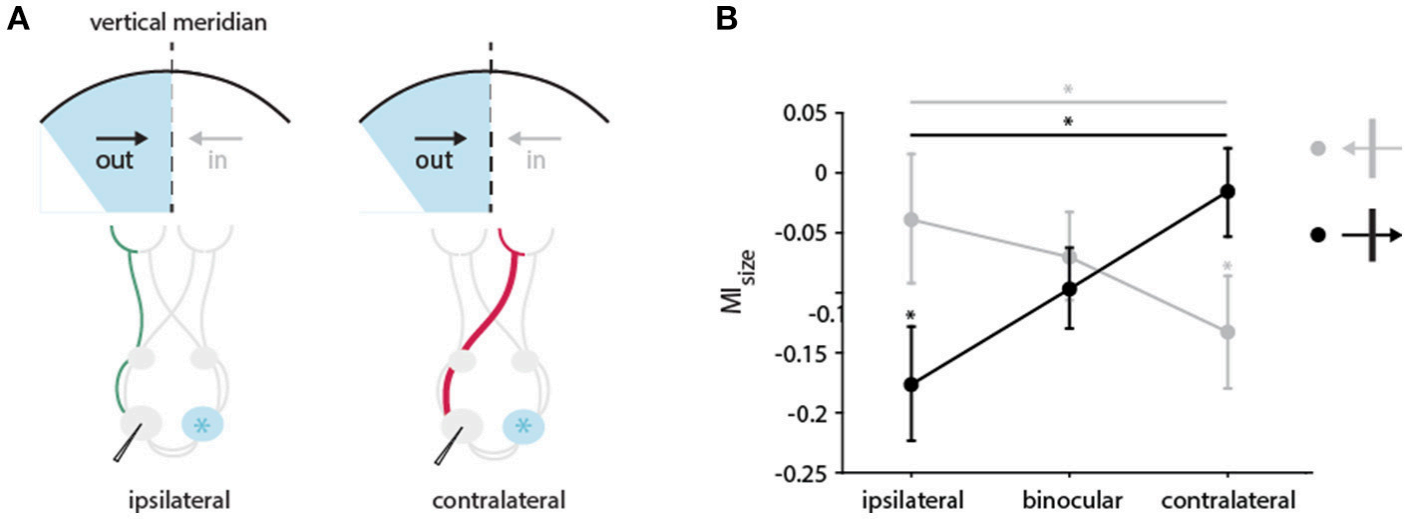
Directional bias in the impact of CC input on receptive field size in the TZ. **(A)** Schematic illustration of the pathways activated through the *ipsi* (in green)-and *contralateral* (in red) eye during cooling of the two groups of neurons preferring either the direction of movement *out of* (black arrow) or *into* (gray arrow) the cooled hemifield (in blue). Cooled hemisphere indicated in blue. **(B)** Mean modulation indices for RF decrease separated by the receiving neuron's direction preference and stimulation of different eyes. Neurons from the two groups preferring either the direction of movement *out of* (black arrow, 0 ± 60 degrees) or *into* (gray arrow, 180 ± 60 degrees) the cooled hemifield are depicted. Stars indicate significant difference between ipsi–and contralateral stimulation (Mann-Whitney-*U*-test, see Results), and baseline and cooling recording (Wilcoxon Signed Rank test, see Results).

To quantify whether this tendency holds for our entire sample including also obliquely preferring neurons we split neurons into two groups, according to their preferred direction of movement (*out of* : 0 ± 60 degrees, *into*: 180 ± 60 degrees). The relationships of the two directions to the recorded hemisphere and the stimulated hemifields are illustrated schematically in Figure 9A. Figure 9B shows the MI_size_ for the two direction groups when stimulated *ipsilaterally, binocularly* or *contralaterally*.

Under *ipsilateral* stimulation, RFs of neurons that prefer direction of movements *into* the cooled hemifield (toward right) are not significantly affected by cooling (*n* = 22, green, left, Wilcoxon signed rank between baseline and cooling, *p* = 0.8), but those of neurons preferring *out of* the cooled hemifield directions (toward left) are (*n* = 28, orange, left, Wilcoxon signed rank between baseline and cooling, *p* = 0.017) as expected. Most interestingly, when stimulating the *contralateral* eye, this finding reverses. The subgroup of RFs preferring *into* directions decreased significantly in size (*n* = 22, green, right, Wilcoxon signed rank between baseline and cooling, *p* = 0.017) whereas the RFs preferring *out of* directions remain unchanged (*n* = 28, orange, right, Wilcoxon signed rank between baseline and cooling, *p* = 0.46).

Accordingly, the effects on MI_size_ with either *ipsilateral* or *contralateral* stimulation are also significantly different between the two directions of motion (Mann-Whitney U, *n* = 50, p_ipsilateral_ = 0.04, p_contralateral_ = 0.03). This directional difference did not at all appear for *binocularly* driven RFs (Figure 9, middle). Apparently, opposite monocular cooling effects on RFs complement each other with binocular stimulation.

The above classification leaves out neurons preferring horizontal contours moving *up* or *down* (*n* = 26, 90 ± 30 degrees, 270 ± 30 degrees), as horizontally preferring neurons are strongly affected independent of their direction of motion (Peiker et al., 2013). Noteworthy, the results in Figure 9B do not change when including this group of neurons into the *out of* group (orange), along the line that horizontal contours potentially cross the VM.

## Discussion

When deactivating visual interhemispheric input, we observed no significant change of the ocular dominance distribution in the TZ and adjacent area 17 and 18. Homogeneous receptive field decreases (and increases) occurred throughout the TZ, especially for ipsilateral stimulation and, to a weaker extent, in nearby area 17 for binocular stimulation, independently of the ocular dominance of the receiving neuron.

Proportionally, RF changes were tiny in comparison to profound firing rate decreases and not related to those. They depended on the orientation and direction preference of the neurons, not on the exact RF positions. In particular, neurons preferring horizontal orientations and movements potentially crossing the VM had their RFs affected.

### Callosal contribution to the ocular dominance distribution in cats

In the absence of callosal input, we observed no significant decay of binocularity in all three subzones investigated. We thus also cannot confirm previous experiments reporting a significant decrease of binocularly driven neurons in the TZ after acute or chronic transection of the corpus callosum, lesion of the contralateral cortex or contralateral cooling.

These early experiments had suggested that callosal afferents contribute the input of the non-chiasm-crossing ipsilateral eye fibers directly to construct binocular neurons close to the VM representation in cats (Berlucchi and Rizzolatti, 1968; Dreher and Cottee, 1975; Payne et al., 1980, 1984; Blakemore et al., 1983; Yinon et al., 1988). Our results are in agreement with tracing data in cats demonstrating that callosal connections link cortical territories innervated by the temporal retina of the same eye (Olavarria, 1996). From this organization, it follows that callosal connections would not contribute to the production of binocular cells in the TZ (Olavarria, 2001). Laing et al. (2015) reported a similar organization for pigmented Long Evans rats, and callosal transection did not affect the binocularity in their striate cortex.

Already Payne et al. (1980) cast doubts on the callosal action on cat binocularity because the loss of binocularity after callosal sectioning was not clearly related to the position of the vertical meridian, and, unexpectedly, the increase in monocularity could be observed until up to 20 degrees eccentricity. Since most of our units were localized around the VM and all within that greater area of Payne et al. (1980), we expected at least some of them to exhibit altered ocular dominance. In contrast, cooling did not lower the incidence of binocularly driven units and neither did it raise the contralateral bias index. Some authors have reported an increase of the post-section binocularity loss with time (Yinon et al., 1988), or concluded that part of it was a result of the surgery-induced trauma (Lepore et al., 1983) together with anesthesia (Minciacchi and Antonini, 1984). This allows the interpretation that ocular dominance changes resulted from long- or short-term plasticity rather than from an immediate and direct loss of transcallosal ipsilateral eye inputs to neurons receiving only direct thalamocortical input from the other contralateral eye. In contrast to the previously used invasive interventions, thermal deactivation only blocks synaptic transmission so that activity within the connective fibers is not affected for a long time (Lomber et al., 1999). Short cooling deactivation of callosal afferents would fall within a window of reversible and unspecific actions, which do not “yet” cause an ocular dominance shift.

It was observed only slightly later that the loss of binocularity was more severe when the animals were younger at transection (Elberger and Smith, 1985) indicating a critical period for ocular dominance plasticity induced via the corpus callosum (see also Elberger, 1984). It was also speculated that the increase in monocularity in animals receiving callosal section could have been caused by them becoming strabismic (Elberger, 1979; see also Minciacchi and Antonini, 1984). The strabismus would result from disturbed binocular interaction executed normally by callosal fibers in the central visual field, not from an immediate loss of ipsilateral eye input caused by intersecting those fibers. As a consequence, strabismus would eventually raise the number of monocular neurons at the expense of binocular ones, especially when intersecting the callosum during the postnatal critical period (Elberger and Smith, 1985).

This interpretation is supported by our result. On the one hand, we see no significant effect on adult ocular dominance distribution with cooling deactivation, but, on the other hand, a pronounced alteration of firing rates evoked by binocular and ipsilateral stimulation. This indicates an interference with binocular and monocular mechanisms acting through CC.

### Callosal contribution to receptive fields

Although a few RFs got completely lost during cooling deactivation, we could not confirm the early reports of receptive fields split in two eye-specific halves at the cortical representation of the vertical meridian (Berlucchi and Rizzolatti, 1968; see Choudhury et al., 1965). Rather than the ipsilateral eye input being literally conveyed via the corpus callosum we observe that callosal fibers exert a facilitating (and sometimes suppressing) effect uniformly increasing (or diminishing) RFs when stimulated through the ipsilateral eye only. This effect could not be explained by the relatively large firing rate decrease (~30% of baseline firing) observed with the mapping stimulus at the same time. First, our calculation of the RF area corrects for rate decrease and, second, RF area and rate changes were not correlated at all. RF shrinkage was also not correlated to initial RF size, but the feature specific effects described above were more pronounced with larger RFs.

There was not a clear relationship to eccentricity, but the effect on RFs driven by ipsilateral eye input dominated in the TZ and adjacent area 17. Interestingly, area 17 fields were affected only when stimulating them through both eyes, pointing toward an input from contralateral binocular neurons.

As already observed with spike rates and cortical maps (Schmidt et al., 2010; Altavini et al., 2017), contralateral thermal deactivation dominantly affected the RFs of neurons preferring cardinally, especially horizontally oriented contours. A closer look revealed cooling induced direction-selective changes among neurons preferring directions crossing the VM. With ipsilateral eye only stimulation, receptive field changes occurred significantly more for neurons preferring the direction of the cooling manipulation (affecting connections from the left to the right hemisphere). This is in agreement with the previously reported ongoing rate decrease of binocularly driven neurons (Peiker et al., 2013), which prefer the direction *out of* the cooled hemifield as if anticipating movement across the midline originating from that hemifield (Figure 9A). Interestingly, the situation almost reversed when stimulating the contralateral eye. Receptive field changes occurred now in the opposite direction of movement as if they would compliment the ipsilateral responses. This makes sense when assuming that also the peripheral monocular segment of the visual field (outermost, not colored 30 degree of the hemifields in Figure 9A) contributes indirectly to the extent of direction-selective RFs close to the midline via the corpus callosum. It would exert this influence by callosal fibers from neurons, which receive direction-selective input from these chiasm-crossing regions of the retina intrahemispherically within the contralateral hemisphere. This input would re-echo indirectly in the RFs affected by callosal input from that hemisphere. As the part of the visual field representation receiving chiasm-crossing input is larger than the part receiving non-chiasm-crossing input, complimentary effects of cooling on RFs stimulated by either eye were to be expected.

### Binocular mechanism mediated through the corpus callosum

In rodents, the majority of retinal fibers cross at the chiasm and each eye dominates in the respective contralateral visual cortex. Here, CC are known to contribute significantly to binocular representations (e.g., Diao et al., 1983; Cerri et al., 2010; but see Laing et al., 2015) and their development (Restani et al., 2009; Pietrasanta et al., 2012).

The functional contribution of CC to binocular mechanisms in carnivores and other animals with frontal eyes remains controversial. Ever since it has been speculated that crossed disparities would have to involve a commissure like the corpus callosum (Mitchell and Blakemore, 1970). At the exact midline of the visual field, farther or closer points to the focal plane fall on either temporal or nasal corresponding retina parts, which get inevitably represented, in different hemispheres. However, early on it was assumed that part of the problem could be solved by the bilaterally represented midline representation (e.g., Tusa et al., 1978; Payne, 1990; White et al., 1999) due to crossed retinal inputs in many frontal-eyed animals (Stone, 1966; Sanderson and Sherman, 1971; Stone et al., 1973; Kirk et al., 1976; Terao et al., 1982). In cats, fine stereopsis in a narrow range of disparities could be easily established by thalamocortical input (Bishop and Henry, 1971; Harvey, 1980).

In agreement with stereopsis being processed in the overlapping representation, it has been noted that callosal section in cats has little effect on stereopsis, but that disparity selective neurons rather depend critically on the retino-geniculo-cortical loop (Lepore et al., 1986). Stereoptic depth perception is preserved in cats that underwent callosal section early, suggesting that although the corpus callosum may be a sufficient pathway for the maintenance of stereopsis in cats, it is not necessary (Timney et al., 1985).

Accordingly, the effects of cooling on binocular RFs could be readily predicted from the sum of the monocular changes for most of the larger RFs close to the VM. However, in particular, for smaller binocular RFs in nearby area 17, the effect of cooling was significantly more pronounced than expected from the sum of the monocular findings alone. This points toward a direct contribution from binocular callosal neurons to those RFs. It is in line with the notion that the transcallosal projection is not eye-specific (Schmidt et al., 1997; Olavarria, 2001), but of equal size for neurons of monocular and binocular eye dominance (McCourt et al., 1990).

### Cardinal bias in callosal connections

Our data indicate that in particular neurons preferring vertical and horizontal contours present diminished RFs in the absence of callosal input. Firstly, this result confirms the anatomical observations that, in cats, CC selectively link neurons preferring iso-orientations (Schmidt et al., 1997; Rochefort et al., 2009). Secondly, it is line with a dominance of cardinal contours that was previously observed in cortical structures and mirrored in the response strength of V1 neurons (cats: Pettigrew et al., 1968; Frégnac and Imbert, 1978; Orban et al., 1981; Leventhal and Schall, 1983; monkeys: Mansfield and Ronner, 1978; Blakemore et al., 1981), the number of neurons (Li et al., 2003), the cortical column size (Chapman and Bonhoeffer, 1998; Coppola et al., 1998; Dragoi et al., 2001; Wang et al., 2003; Yacoub et al., 2008), as well as in the maps of ongoing activity (Kenet et al., 2003). Our present finding confirms our previous studies supporting that the interhemispheric network exhibits a cardinal bias in its ongoing (Altavini et al., 2017) and stimulus-driven actions (Schmidt et al., 2010). Here, we extend this action to the construction of RFs in the area receiving callosal input. For the first time, we describe a cardinal bias for the contribution of lateral connectivity to RFs. Interestingly, the influence on the RFs was not correlated with the overall strength of excitatory input reflected in the loss of firing rate during cooling and smaller in its relative impact, and thus not explained by it. Previously, the firing rate loss was shown to consist mainly in a multiplicative scaling of the response, which can occur in a mixture with a smaller additive component of different size (Wunderle et al., 2013). As the RF size estimate largely corrects for overall firing rate changes we assume that the larger multiplicative component contributes less to RF shrinkage. Moreover, RF changes were not equally expressed in all neurons, which were affected in their spike rate. Therefore, we consider the possibility that these changes were related to the smaller additive contribution of callosal input. The latter component supposedly subtracts a certain number of spikes from all responses, is not correlated with the size of the multiplicative component, and its impact varied between neurons and stimulations.

Westheimer (2003) proposed the oblique effect as an innate organizational feature in the visual system facilitating visual processing along vertical and horizontal lines. Our results fortify this notion because the receptive field is a neuronal feature emergent from geniculo-cortical input and intracortical circuits.

In concordance with the effects observed on ongoing activity (Peiker et al., 2013), RF changes mirrored the direction of the cooling procedure (for left to right) affecting monocular responses in a direction-selective way. This led us conclude that lateral interhemispheric—and probably also intracortical connections—implement priors into the cortical network for anticipating cardinal contours and movements which are likely to cross the VM. These priors become more obvious especially when sensory input is less salient (Patten et al., 2017), namely in the responses to the ipsilateral eye and in ongoing activity.

## Author contributions

KS designed the experiment, conducted the research, analyzed the data and wrote the manuscript. SC-O conducted the research, analyzed the data, and wrote the manuscript. CJ designed the experiment and conducted the research. TW and DE conducted the research and provided analysis tools, SN provided analysis tools, acquisition tools and experimental setup. All authors contributed to the manuscript.

### Conflict of interest statement

The authors declare that the research was conducted in the absence of any commercial or financial relationships that could be construed as a potential conflict of interest.
